# The clinical association of left atrial appendage thrombus on CTA with functional outcome

**DOI:** 10.1093/esj/23969873251377215

**Published:** 2026-01-01

**Authors:** Joel Winders, Angelo Di Bartolo, Jamin Kim, Duncan Wilson, Sajith Senadeera, Yassar Alamri, John Fink, James Beharry, Mark W Parsons, Christopher Levi, Neil Spratt, Beng Lim Alvin Chew, Md Golam Hasnain, Ferdinand Miteff, Leon Rinkel, Shan Sui Nio, Sinan Al-Hadethi, Anthony Lim, Jonathan Coutinho, Carlos Garcia-Esperon, Teddy Y Wu, Alexander Berry-Noronha

**Affiliations:** Department of Neurology, Christchurch Hospital, Christchurch, New Zealand; Department of Radiology, Christchurch Hospital, Christchurch, New Zealand; Department of Neurology, Christchurch Hospital, Christchurch, New Zealand; New Zealand Brain Research Institute, Christchurch, New Zealand; Department of Radiology, Christchurch Hospital, Christchurch, New Zealand; Department of Neurology, Christchurch Hospital, Christchurch, New Zealand; Department of Neurology, Christchurch Hospital, Christchurch, New Zealand; Department of Neurology, Christchurch Hospital, Christchurch, New Zealand; Department of Neurology, John Hunter Hospital, Newcastle, NSW, Australia; Sydney Brain Centre, Ingham Institute for Applied Medical Research, Liverpool, NSW, Australia; Faculty of Medicine, University of Newcastle, Callaghan, NSW, Australia; Hunter Medical Research Institute, Newcastle, NSW, Australia; Department of Neurology, John Hunter Hospital, Newcastle, NSW, Australia; Faculty of Medicine, University of Newcastle, Callaghan, NSW, Australia; Hunter Medical Research Institute, Newcastle, NSW, Australia; Department of Neurology, John Hunter Hospital, Newcastle, NSW, Australia; Faculty of Medicine, University of Newcastle, Callaghan, NSW, Australia; Hunter Medical Research Institute, Newcastle, NSW, Australia; Department of Neurology, John Hunter Hospital, Newcastle, NSW, Australia; Faculty of Medicine, University of Newcastle, Callaghan, NSW, Australia; Hunter Medical Research Institute, Newcastle, NSW, Australia; Department of Neurology, John Hunter Hospital, Newcastle, NSW, Australia; Department of Neurology, Amsterdam UMC, University of Amsterdam, Amsterdam, The Netherlands; Department of Neurology, Amsterdam UMC, University of Amsterdam, Amsterdam, The Netherlands; Department of Radiology, John Hunter Hospital, Newcastle, NSW, Australia; Department of Radiology, Christchurch Hospital, Christchurch, New Zealand; Department of Neurology, Amsterdam UMC, University of Amsterdam, Amsterdam, The Netherlands; Department of Neurology, John Hunter Hospital, Newcastle, NSW, Australia; Faculty of Medicine, University of Newcastle, Callaghan, NSW, Australia; Hunter Medical Research Institute, Newcastle, NSW, Australia; Department of Neurology, Christchurch Hospital, Christchurch, New Zealand; Department of Medicine, The University of Otago, Christchurch, New Zealand; Department of Neurology, Christchurch Hospital, Christchurch, New Zealand; Department of Medicine, The University of Melbourne, Melbourne, VIC, Australia

**Keywords:** Stroke, left atrial appendage thrombus, atrial fibrillation

## Abstract

**Background:**

Left atrial appendage (LAA) thrombus is associated with atrial fibrillation (AF) and can be a marker of atrial cardiomyopathy. We determined the association between computed tomography angiography (CTA) identified LAA thrombus in patients presenting with acute ischaemic stroke or transient ischaemic attack (TIA), and 3-month outcome.

**Methods:**

We undertook a dual-centre, retrospective cohort study from New Zealand and Australia. All consecutive patients presenting with acute ischaemic stroke or TIA during the inclusion period who underwent acute stroke imaging were included. We analysed the association with CTA-LAA thrombus and 3-month outcome on modified Rankin Scale using multivariable logistic regression models adjusted for known predictors of outcome.

**Results:**

Of the 1435 patients included, 1304 (90.9%) had acute ischaemic stroke and 131 (9.1%) had TIA. 582 (41%) had confirmed intracranial medium or large vessel occlusion (MLVO), and 565 (40%) received reperfusion therapies. CTA-LAA thrombus was identified in 58 (4.0%) patients, and these patients were older (median age 85 (IQR 75–88) vs 73 (63–81), *p* < 0.01), more likely to be female (62% vs 40%, *p* < 0.01), had higher rates of AF (79% vs 29%, *p* < 0.01), heart failure (29% vs 9%, *p* < 0.01), MLVO (53% vs 40%, *p* = 0.05), and mortality at 3-months (28% vs 11%, *p* < 0.01). Adjusting for known predictors of poor outcome, LAA thrombus was independently associated with increased 3-month mRS score (OR: 2.02, 95% CI: 1.20–3.40, *p* < 0.01).

**Conclusions:**

CTA-LAA thrombus detected during the acute stroke imaging protocol in patients with ischemic stroke or TIA is a predictor of worse outcome.

## Graphical Abstract

**Graphical abstract:**
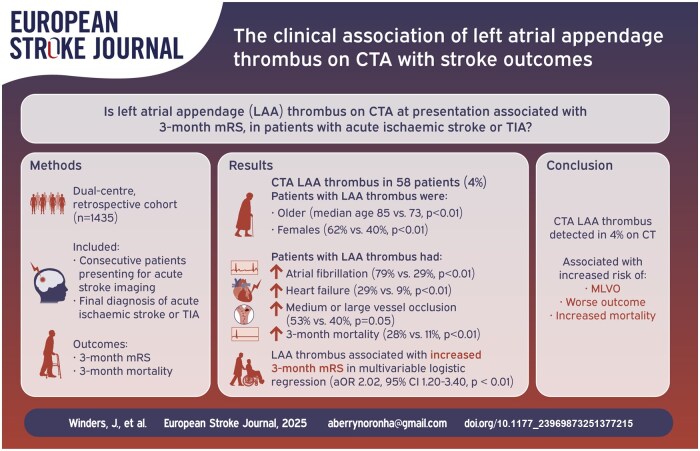

## Background

A cardiac source of embolus accounts for approximately 30% of ischaemic strokes and is associated with a higher burden of disability and mortality compared to other causes of stroke.^1^ One potential mechanism is thrombus formation in the left atrial appendage (LAA), as occurs in the presence of atrial fibrillation (AF) and atrial cardiomyopathy.^2,3^ Cardiac computed tomography (CT) is an imaging modality that has been adopted as part of routine hyperacute stroke imaging in several comprehensive stroke centres internationally, and may overcome issues with access to transthoracic echocardiography (TTE). It has been previously demonstrated that cardiac CT in hyperacute stroke has superior yield to TTE for identifying cardiac source of emboli.^4^ In our previous study, CT angiography (CTA) LAA thrombus was present in 6.6% of patients with ischaemic stroke, and was associated with known or newly diagnosed AF.^5^

The acute stroke pathway CT imaging sequences at Christchurch Hospital, Christchurch, New Zealand, and John Hunter Hospital, Newcastle, Australia (JHH), includes non-contrast CT, CT perfusion and CTA from the LAA to vertex.^6^ This is a non-invasive method of detecting LAA thrombus,^7–13^ which may serve as a marker of atrial cardiomyopathy, prolonging the scan by 30 s–2 min, without needing additional contrast.^5^ The Christchurch and JHH protocols result in an estimated increased radiation of 1.2 and 3.7 mSv respectively. A recent randomised trial comparing standard acute stroke CTA imaging with extended CTA imaging 6 cm below the carina showed significantly higher rates of cardioaortic thrombus detection in the extended CTA group (8.8% vs 1.7%, *p* = 0.02) without compromising time to completion of imaging.^14^ The presence of LAA thrombus may confer increased early recurrent ischaemic stroke risk, although there are no published studies addressing this as a primary outcome. LAA thrombus may also indicate undetected AF in patients without prior history of AF, enabling early anticoagulation and abrogate the need for resource-intensive cardiac rhythm monitoring. In patients with known AF, identification of LAA thrombus is also valuable as it may highlight compliance issues, or prompt consideration of change in treatment. The association between LAA thrombus detected with CTA at the time of acute stroke presentation and clinical outcome has not been fully explored. Our aim was to investigate this association, hypothesising that presence of CTA-LAA thrombus at the time of acute stroke imaging would be associated with higher morbidity and mortality.

## Methods

We performed a retrospective analysis to investigate the association between CTA-LAA thrombus detected at time of initial cerebrovascular presentation with 3-month functional outcome.

### Patients

We included all consecutive patients presenting to the emergency department with stroke-like symptoms who would have been potential candidates for acute thrombolysis or mechanical thrombectomy (MT). This typically included patients with potentially disabling deficit (Christchurch), or NIHSS ⩾ 4 (JHH).^15^ all of whom underwent multimodal brain imaging (non-contrast CT, CT perfusion and CTA) and cardiac CT at two comprehensive stroke centres in New Zealand (Christchurch) and Australia (JHH) between September 2018 and December 2020, and November 2020 and August 2023, respectively. We excluded patients where the final diagnosis was not ischaemic stroke or transient ischaemic attack (TIA). Clinical data were extracted from comprehensive electronic clinical records and review of radiological imaging. We obtained clinical variables including baseline demographics, evidence of previous or current AF, reperfusion treatment, demographic risk factors and examined for presence of LAA thrombus, and medium or large vessel occlusion (MLVO). Three-month modified Rankin Scale (mRS) was determined from detailed electronic clinical records at 3-month follow up, as undertaken by the stroke rehabilitation team. MLVO was defined as CTA occlusion of the extracranial or intracranial internal carotid artery (ICA); anterior cerebral artery (ACA); first, second or third divisions of the middle cerebral artery (M1, M2 and M3 respectively); vertebral or basilar arteries. Ischaemic stroke was defined as an acute neurological deficit accompanied by evidence of acute ischaemia on imaging (CT or magnetic resonance imaging). TIA was defined as an acute neurological deficit lasting less than 24 h, with normal acute and follow-up imaging and no other explanation for the presentation. Recurrent ischaemic stroke was defined accordingly, with information recorded from hospital records. Recurrent embolic events included myocardial infarction or any diagnosis of thromboembolic events in hospital records.

### Statistical analysis

Baseline demographics and risk factors were assessed using Shapiro–Wilk test for normality. These were then compared with *p*-values obtained using the Wilcoxon rank test, while binary variables were compared using the Chi-squared method. Where less than 10 values were present, Fisher’s exact test was used. To assess the association with LAA thrombus and 3-month outcome (mRS 0–6), we undertook a multivariable ordered logistic model adjusting for known predictors of poor outcome as fixed effects (age, premorbid mRS, NIHSS, MLVO, congestive heart failure, AF and treatment with MT or intravenous thrombolysis). A subgroup analysis was also undertaken on a cohort that excluded patients with TIAs. Statistical analysis was performed using RStudio 2023.03.0.

### Brain imaging protocol

The Christchurch imaging protocol consists of non-contrast CT brain, followed by CT perfusion and extended range CTA from the left atrium to the vertex. All patients were examined using either a 128-slice (SOMATOM Definition Flash, Siemens Healthcare, Erlangen, Germany) or 64-slice (GE VCT Lightspeed, GE Healthcare, Milwaukee, WI, USA) CT scanner. The JHH imaging protocol consisted of non-contrast CT brain, followed by CT perfusion, and then CTA from aortic arch to skull vertex after 5 min. CTA of the left atrium was performed at 30 s and 2 min. All patients were examined using a 384 (2 × 192) slice Dual Source CT scanner (SOMATOM Force, Siemens Healthcare, Erlangen, Germany or GE VCT Lightspeed, GE Healthcare, Milwaukee, WI, USA). CTA was not cardiac-gated at either site. See the Supplementary Appendix S1 for more detailed protocols.

### Radiological analysis

CTA review was undertaken by independent radiology investigators at respective sites (AD and SS at Christchurch; CG, BC and SA at JHH) with images read by the clinical research team.

**Figure 1.**
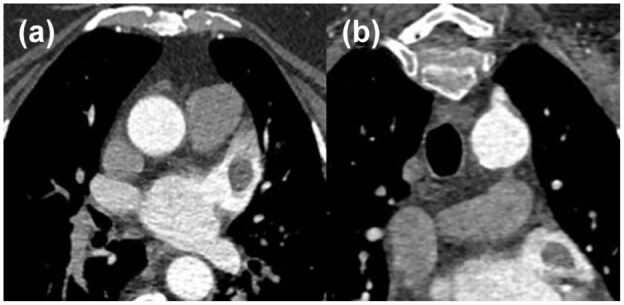
Positive CTA imaging demonstrating free-floating LAA thrombus on axial (a) and coronal (b) sequences.

**Figure 2.**
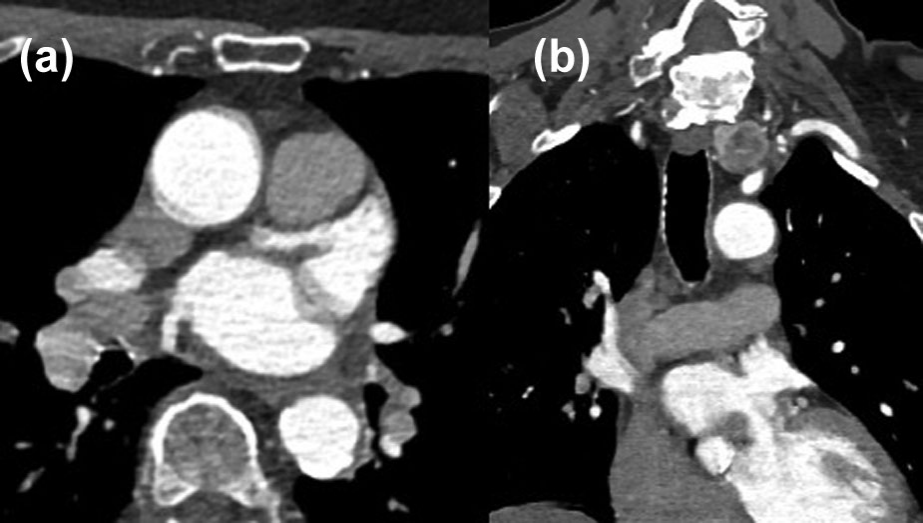
CTA imaging negative for LAA thrombus on axial (a) and coronal (b) sequences.

**Figure 3.**
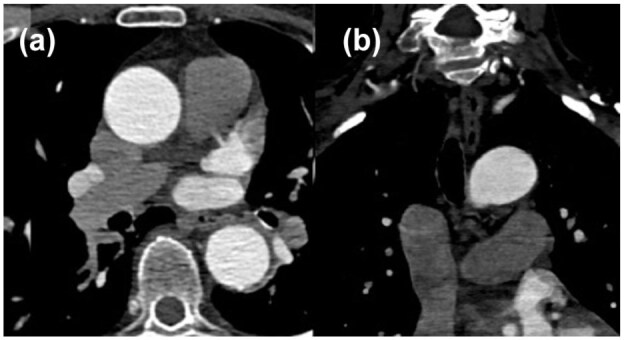
Indeterminate CTA imaging for LAA thrombus on axial (a) and coronal (b) sequences.

The following criteria for determining likely presence of LAA thrombus were used:

*Positive:* Filling defect not caused by motion artefact or atrial trabeculae that has Hounsfield units (HU) < 100 AND oval/rounded in shape with well-defined border (Figure 1).
*Negative:* LAA completely opacifies with contrast, except of changes that can be explained by motion artefact or atrial trabeculae (Figure 2).
*Indeterminate:* Not fulfilling criteria for positive or negative (Figure 3). This includes cases of slow-flow in the LAA.

Indeterminate results were reviewed with a second reader (AL at Christchurch, and SA radiologist with cardiac fellowship at JHH) and either resolved by mutual agreement or were recorded as indeterminate and excluded from the final analysis. This included cases of slow-flow to the LAA. Delayed phase CTA was used when available to clarify if necessary.

### Ethics approval

For the Christchurch patients, data were taken from the Christchurch Stroke Registry study and have full approval from the National Health Disability Committee (application reference 17/CEN/84). For the JHH patients, data were retrieved from the International Stroke Perfusion Imaging Registry (INSPIRE), with ethics approval by the Hunter New England Local Health District Human Research Ethics Committee in accordance with Australian National Health and Medical Research Council guidelines (Reference No: 11/08/17/4.01).

## Results

**Figure 4.**
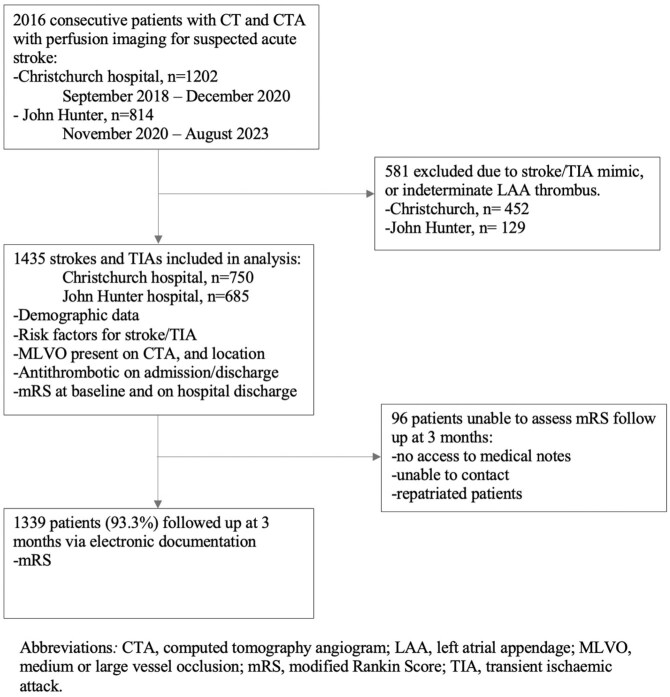
Flow diagram of patient recruitment to follow-up.

A total of 2016 patients presenting with stroke-like symptoms underwent multimodal brain imaging with hyperacute cardiac CT across the two sites during the inclusion period. Of them, 523 patients were not ultimately diagnosed with a stroke or TIA; 58 patients had a non-diagnostic cardiac CT (due to movement artefacts or an indeterminate filling defect), and 96 had no 3-month outcome data available, leaving a total of 1339 included in this study (Figure 4). A final diagnosis of acute ischaemic stroke was diagnosed in 1304 (90.9%) patients, and 131 (9.1%) were diagnosed as TIA. The median age was 74 (IQR: 63–82) years, 589 (41%) were female sex, 582 (41%) had confirmed intracranial MLVO, and 565 (40%) received any type of hyperacute reperfusion therapy (Table 1).

**Table 1.** Baseline demographics of cohorts and 3-month outcomes.

Variable	Total (*n* = 1435)	LAA present (*n* = 58)	LAA not present (*n* = 1377)	*p*-Value
Age median (IQR)	74 (63–82)	85 (75–88)	73 (63–81)	<0.01
Sex-female (%)	589 (41%)	36 (62%)	553 (40%)	<0.01
Hypertension	825 (58%)	32 (55%)	793 (58%)	0.97
Diabetes	301 (21%)	12 (21%)	289 (21%)	1.00
Dyslipidaemia	405 (28%)	14 (24%)	391 (28%)	0.65
Atrial fibrillation	439 (31%)	46 (79%)	393 (29%)	<0.01
Ischaemic heart disease	292 (20%)	13 (22%)	279 (20%)	0.67
Heart failure	141 (10 %)	17 (29%)	124 (9%)	<0.01
Peripheral vascular disease	137 (10%)	7 (12%)	130 (9%)	0.68
Previous stroke	252 (18%)	13 (22%)	239 (17%)	0.33
MLVO (%)	582 (41%)	31 (53%)	551 (40%)	0.05
Thrombolysis	333 (23%)	17 (29%)	316 (23%)	0.16
MT	333 (23%)	22 (38%)	311 (23%)	0.01
Any reperfusion treatment	565 (40%)	28 (48%)	536 (39%)	0.11
3-month outcomes				
3-month mortality (%)	169 (12%)	16 (28%)	153 (11%)	<0.01
mRS, median (IQR)	2 (1–4)	3 (2–6)	2 (1–3)	<0.01
Recurrent ischaemic stroke	50 (3%)	5 (9%)	45 (3%)	0.05
Recurrent embolic event	60 (4%)	6 (10%)	54 (4%)	0.03

IQR: interquartile range; MLVO: medium or large vessel occlusion; mRS: modified Rankin Scale; MT: mechanical thrombectomy.

LAA thrombus was present in 58 (4.0%) of patients, all of whom had a final diagnosis of ischaemic stroke. In comparison to patients without LAA thrombus, patients with LAA thrombus were older (85 vs 73 years, *p* < 0.01), more likely to be female (62% vs 40%, *p* < 0.01), had a higher rate of AF (79% vs 29%, *p* < 0.01) and heart failure (29% vs 9.0%, *p* < 0.01). Rates of hypertension, diabetes, dyslipidaemia, ischaemic heart disease, peripheral vascular disease, thrombolysis and any reperfusion treatment were similar between groups, though patients with LAA thrombus had increased rate of MT (38% vs 23%, *p* = 0.01). Patients with LAA thrombus had a higher rate of MLVO (53% vs 40%) but this did not reach statistical significance (*p* = 0.05; Table 1).

Patients with a final diagnosis of ischaemic stroke or TIA and presence of LAA thrombus in the hyperacute cardiac CT imaging had increased 3-month mortality (28% vs 11%, *p* < 0.01) and worse functional outcome (3-month mRS score of 3 (2–6) vs 2 (1–3), *p* < 0.01).

In our multivariable ordered logistic model adjusting for known predictors of poor outcome, LAA thrombus was associated with increased odds of morbidity, based on higher 3-month mRS scores (aOR: 2.01, 95% CI: 1.20–3.40, *p* = 0.01; Table 2). Other variables associated with increased morbidity in this model were age-increase by 1-year (1.04 (1.03–1.04), *p* < 0.01) and MLVO (2.11 (1.67–2.67), *p* < 0.01), while treatment with thrombolysis (0.74 (0.59–0.93), *p* = 0.01) and MT (0.67 (0.50–0.89), *p* < 0.01) were associated with reduced morbidity. These findings did not differ with exclusion of TIAs from the analysis.

**Table 2.** Association between risk factors and increased 3-month mRS score, as determined from our multivariable ordered logistic model, from our full cohort, and a cohort that excluded TIAs.

Risk factor	Full cohort		TIAs excluded	
	Odds ratio (95% CI)	*p*-Value	Odds ratio (95% CI)	*p*-Value
Age (increase in 1 year)	1.04 (1.03–1.04)	<0.01	1.04 (1.03–1.05)	<0.01
Premorbid mRS (increase by 1)	1.66 (1.48–1.85)	<0.01	1.60 (1.43–1.79)	<0.01
NIHSS (increase by 1)	1.07 (1.05–1.09)	<0.01	1.07 (1.05–1.09)	<0.01
MLVO	2.11 (1.67–2.67)	<0.01	1.86 (1.47–2.36)	<0.01
LAA thrombus	2.02 (1.20–3.40)	<0.01	1.99 (1.17–3.39)	0.01
Heart failure	1.28 (0.91–1.78)	0.15	1.28 (0.91–1.81)	0.16
Atrial fibrillation	1.09 (0.86–1.37)	0.28	1.07 (0.84–1.36)	0.50
Thrombolysis	0.74 (0.58–0.93)	0.01	0.64 (0.50–0.81)	<0.01
MT	0.67 (0.50–0.89)	<0.01	0.65 (0.49–0.87)	<0.01

LAA: left atrial appendage; MLVO: medium or large vessel occlusion; mRS: modified Rankin Scale; MT: mechanical thrombectomy.

## Discussion

In our study, the presence of CTA-LAA thrombus detected during acute stroke imaging was both independently associated with 3-month mortality and associated with increased 3-month mRS. Our results suggest CTA-LAA thrombus can be used as an important biomarker for risk and prognosis.

The independent association between LAA thrombus and increased 3-month mortality and morbidity substantiates preliminary findings from previous literature, where stroke patients with LAA thrombus have been shown to have more severe baseline stroke, a higher risk of cardiovascular death (RR: 5.52) and poor clinical outcome defined as mRS score of >3 (RR: 3.73).^16,17^ There are several possible mechanisms accounting for the increased morbidity in stroke patients with CTA-LAA thrombus. Firstly, the association in our study between CTA-LAA and MLVO suggests that these patients are more likely to have larger infarcts with more severe baseline strokes, translating to worse functional outcome or increased risk of mortality. Secondly, LAA thrombus could be a marker of underlying cardiac disease, which can contribute to poorer outcomes. Thirdly, patients with LAA thrombus are older; however we found this association independent of age. Importantly, our study demonstrates these associations in an acute stroke population not limited to patients with AF. The combination of increased morbidity and mortality outcomes suggests that CTA-LAA thrombus can be used as a radiological biomarker of clinical prognosis.

Our results contrast with those from a recent study of 370 acute stroke and TIA patients, where a higher mRS score in patients with CTA-LAA thrombus (3 (1–6) vs 2 (1–5), *p* = 0.11) was not statistically significantly.^8^ This may be attributed to smaller sample size, with our results being further validated by inclusion of multiple centres, reducing bias. It is notable that this study found a higher rate of LAA thrombus (9%) than both our study (4%) and other similar studies (6.7%).^3^ This higher rate could be attributable to differences in imaging techniques used, such as spectral CT reconstructions and iodine maps, potentially increasing diagnostic sensitivity compared to CTA. A change in protocol in our study resulted in 465 patients undergoing non-delayed imaging, which may have contributed to under-detection. It is also possible that non-uniformity in CTA protocols between centres and a retrospective design may have contributed to variability in our study results. Other differences include an older median patient age in our cohort (67 years vs 60), and a lower rate of intravenous thrombolysis in our study (23% vs 36%), though these would not necessarily explain the difference in association between LAA thrombus and outcome.

It is notable that the rate of LAA thrombus was different between our two sites (1.7% in JHH and 6.0% in Christchurch). This difference may be attributable to population differences between the cohorts, including a lower median age (73 (61–80) vs 75 (66–83)) and a higher rate of anticoagulation in the Australian cohort (16% vs 12.5%) despite a lower rate of AF (26% vs 35%) and lower rate of heart failure (8% vs 11%). This difference may also be influenced by differences in local protocols, with Christchurch using stroke severity as the threshold for undertaking hyperacute CT scan, which may selected for higher severity strokes. The lower rate of AF in the JHH cohort is likely the main contributing factor for the lower CTA LAA detection rate between the two groups.

The higher rates of recurrent ischaemic stroke and embolic events in patients with LAA thrombus is hypothesis generating. While this has been explored with transoesophageal echocardiography in non-stroke populations previously, an association between LAA thrombus and recurrent events has not been consistently demonstrated.^16,18,19^ Due to the low overall recurrent event numbers in our study, this should be considered exploratory and validated in larger, prospective studies.

Validating the use of CTA for left atrial or LAA thrombus identification was not an aim of this study, but CTA has the advantage of being non-invasive, and can be undertaken at the time of an acute stroke imaging protocol. In centres where transoesophageal echocardiogram is not commonly performed, LAA thrombi may not be identified in stroke patients which may lead to an incorrect diagnosis of embolic stroke of undetermined source (ESUS), or cryptogenic stroke. Recent ESUS trials^20–22^ assessed the efficacy of oral anticoagulation for secondary stroke prevention in those classified as ESUS or cryptogenic stroke. These trials failed to show a clinical benefit of oral anticoagulation in secondary stroke prevention. The classification of ESUS or cryptogenic stroke in these trials did not include dedicated LAA imaging to investigate a cardioembolic source of stroke, thus potentially missing a biomarker for embolic stroke, which may have masked treatment benefit. Future studies could investigate enriched ESUS cohorts with LAA thrombus assessment to determine the benefit of prolonged cardiac monitoring or anticoagulation.

There were several strengths to our study. This one of the largest studies to focus on the relationship between LAA thrombus in patients presenting with acute stroke/TIA, and their clinical outcomes. We included all consecutive patients undergoing acute stroke imaging within the study timeframe. Inclusion of two centres improved the generalisability of our results at an international level. Finally, the availability of 3-month follow-up data was high (93.3%).

Our study also had limitations. Our patient cohort is selected based on centre-specific practices that determine selection for acute stroke imaging. In terms of imaging, CT was not cardiac-gated in either site during the study period. Furthermore, in Christchurch a change in protocol during the study period meant that the initial 465 patients underwent only non-delayed CTA, which may have reduced sensitivity of diagnosis of LAA thrombus. It is also recognised that the imaging protocols between the two sites, whilst similar, were not identical, with interpretation by separate radiologists, which may have impacted detection rates. Radiologists were not strictly blinded in this study, however the interpretation of LAA thrombus was in isolation to the overall CT imaging and clinical outcome, thus limiting risk for bias. Data collection was non-contemporaneous between the two sites, with Christchurch completing their recruitment a month after JHH started recruiting in 2020, but work-flow between the centres was similar.^23^ The primary outcome, mRS, may be subject to inter-observer variability which may have impacted our results. This is perhaps further impacted through the retrospective collection from medical records, as well as influence of prior functional status (noting that 18% of our cohort had a previous stroke). Imaging beyond the LAA was not available in this study, thus some patients with alternate cardioembolic aetiologies (such as left ventricular thrombi) may not be identified. Known history of AF and new AF at presentation were not distinguished. Data on post-stroke antiplatelets and anticoagulant therapy were not available for all patients, thus limiting our analysis of recurrent ischaemic stroke or embolic events.

## Conclusions

Our results suggest LAA thrombus identified on acute stroke CTA imaging has potential to be an easily obtainable and clinically meaningful prognostic biomarker of morbidity and mortality. The role of CTA-LAA thrombus in recurrent ischaemic stroke or embolic events needs to be investigated in future larger, prospective studies.

## Supplementary Material

sj-docx-1-eso_23969873251377215

## Data Availability

Further data is available from the authors on request.

## References

[1] Arboix A, Alioc J. Cardioembolic stroke: clinical features, specific cardiac disorders and prognosis. Curr Cardiol Rev 2010; 6(3): 150–161. 21804774 10.2174/157340310791658730 PMC2994107

[2] Kaarisalo MM, Immonen-Räihä P, Marttila RJ, et al. Atrial Fibrillation and stroke: mortality and causes of death after the first acute ischemic stroke. Stroke 1997; 28(2): 311–315. 9040681 10.1161/01.str.28.2.311

[3] Nguyen HT, Nguyen HVB, Nguyen HQ, et al. Prevalence of left atrial appendage thrombus in patients with acute ischaemic stroke and sinus rhythm: a cross-sectional study. BMJ Open 2021; 11(12): e051563. 10.1136/bmjopen-2021-051563 PMC8685935 34921077

[4] Rinkel LA, Guglielmi V, Beemsterboer CFP, et al. Diagnostic yield of ECG-gated cardiac CT in the acute phase of ischemic stroke vs transthoracic echocardiography. Neurology 2022; 99(14): e1456–e1464. 10.1212/WNL.0000000000200995 35918169

[5] Senadeera SC, Palmer DG, Keenan R, et al. Left atrial appendage thrombus detected during hyperacute stroke imaging is associated with atrial fibrillation. Stroke 2020; 51(12): 3760–3764. 33161849 10.1161/STROKEAHA.120.030258

[6] Wu TY, Coleman E, Wright SL, et al. Helsinki stroke model is transferrable with “real-world” resources and reduced stroke thrombolysis delay to 34 min in Christchurch. Front Neurol 2018; 9: 290. 29760676 10.3389/fneur.2018.00290 PMC5937050

[7] Hur J, Kim YJ, Lee HJ, et al. Left atrial appendage thrombi in stroke patients: detection with two-phase cardiac CT angiography versus transesophageal echocardiography. Radiology 2009; 251(3): 683–690. 19366905 10.1148/radiol.2513090794

[8] Kauw F, Velthuis BK, Takx RAP, et al. Detection of cardioembolic sources with nongated cardiac computed tomography angiography in acute stroke: results from the ENCLOSE study. Stroke 2023; 54(3): 821–830. 36779342 10.1161/STROKEAHA.122.041018 PMC9951793

[9] Kim YY, Klein AL, Halliburton SS, et al. Left atrial appendage filling defects identified by multidetector computed tomography in patients undergoing radiofrequency pulmonary vein antral isolation: a comparison with transesophageal echocardiography. Am Heart J 2007; 154(6): 1199–1205. 18035095 10.1016/j.ahj.2007.08.004

[10] Kitayama H, Kiuchi K, Endo T, et al. Value of cardiac ultrafast computed tomography for detecting right atrial thrombi in chronic atrial fibrillation. Am J Cardiol 1997; 79(9): 1292–1295. 9164911 10.1016/s0002-9149(97)00107-0

[11] Love BB, Struck LK, Stanford W, et al. Comparison of two-dimensional echocardiography and ultrafast cardiac computed tomography for evaluating intracardiac thrombi in cerebral ischemia. Stroke 1990; 21(7): 1033–1038. 2368104 10.1161/01.str.21.7.1033

[12] Shapiro MD, Neilan TG, Jassal DS, et al. Multidetector computed tomography for the detection of left atrial appendage thrombus: a comparative study with transesophageal echocardiography. J Comput Assist Tomogr 2007; 31(6): 905–909. 18043355 10.1097/rct.0b013e31803c55e3

[13] Tomoda H, Hoshiai M, Furya H, et al. Evaluation of intracardiac thrombus with computed tomography. Am J Cardiol 1983; 51(5): 843–852. 6829443 10.1016/s0002-9149(83)80143-x

[14] Sposato LA, Ayan D, Ahmed M, et al. Extended CT angiography versus standard CT angiography for the detection of cardioaortic thrombus in patients with ischaemic stroke and transient ischaemic attack (DAYLIGHT): a prospective, randomised, open-label, blinded end-point trial. Lancet Neurol 2025; 24(6): 489–499. 40409313 10.1016/S1474-4422(25)00111-5

[15] Garcia-Esperon C, Berry-Noronha A, Di Bartolo A, et al. Arterial input function dispersal on acute brain CT perfusion scan in patients with acute stroke and an intracardiac thrombus. Neurology 2025; 104(3): e210256. 10.1212/WNL.0000000000210256 39805052

[16] Dawn B, Varma J, Singh P, et al. Cardiovascular death in patients with atrial fibrillation is better predicted by left atrial thrombus and spontaneous echocardiographic contrast as compared with clinical parameters. J Am Soc Echocardiogr 2005; 18(3): 199–205. 15746706 10.1016/j.echo.2004.12.003

[17] Heo J, Lee H, Lee IH, et al. Impact of left atrial or left atrial appendage thrombus on stroke outcome: a matched control analysis. J Stroke 2023; 25(1): 111–118. 36592972 10.5853/jos.2022.02068 PMC9911853

[18] Stoddard MF, Singh P, Dawn B, et al. Left atrial thrombus predicts transient ischemic attack in patients with atrial fibrillation. Am Heart J 2003; 145(4): 676–682. 12679765 10.1067/mhj.2003.91

[19] Stöllberger C, Chnupa P, Abzieher C, et al. Mortality and rate of stroke or embolism in atrial fibrillation during long-term follow-up in the embolism in left atrial thrombi (ELAT) study. Clin Cardiol 2004; 27(1): 40–46. 14743856 10.1002/clc.4960270111 PMC6654077

[20] Diener HC, Sacco RL, Easton JD, et al. Dabigatran for prevention of stroke after embolic stroke of undetermined source. N Engl J Med 2019; 380(20): 1906–1917. 31091372 10.1056/NEJMoa1813959

[21] Hart RG, Sharma M, Mundl H, et al. Rivaroxaban for stroke prevention after embolic stroke of undetermined source. N Engl J Med 2018; 378(23): 2191–2201. 29766772 10.1056/NEJMoa1802686

[22] Kamel H, Longstreth WT Jr, Tirschwell DL, et al. Apixaban to prevent recurrence after cryptogenic stroke in patients with atrial cardiopathy: the ARCADIA randomized clinical trial. JAMA 2024; 331(7): 573–581. 38324415 10.1001/jama.2023.27188 PMC10851142

[23] Garcia-Esperon C, Wu TY, Carraro do Nascimento V, et al. Ultra-long transfers for endovascular thrombectomy—mission impossible? The Australia-New Zealand experience. Stroke 2023; 54(1): 151–158. 36416128 10.1161/STROKEAHA.122.040480

