# Supplementary material for: The clinical association of left atrial appendage thrombus on CTA with functional outcome
Source: Eur Stroke J. 2026 Jan 1;11(1):23969873251377215. doi: 10.1093/esj/23969873251377215 (PMC12866231; doi:10.1093/esj/23969873251377215)
Supplement: sj-docx-1-eso_23969873251377215 [file sj-docx-1-eso_23969873251377215.docx]

**Supplementary appendix S1:** Detailed description of Christchurch and John Hunter Hospital imaging protocols:

Christchurch: All patients were examined using either a 128 or 64-slice MDCT scanner (SOMATOM Definition Flash, Siemens Healthcare, Forchheim, Germany or GE VCT Lightspeed, GE Healthcare, Waukesha, WI, USA). SOMATOM scan parameters for the CTP were using 128×0.6mm collimation, 0.285 s rotation time, 70 kV tube voltage and 250mAs effective tube current time product. Contrast enhanced CT perfusion scans were acquired using 40 ml Omnipaque 300 (GE Healthcare, WI, USA), 30ml bolus sodium chloride and an injection rate of 6 ml/s. A soft spatial resolution kernel (H20f) was chosen to reconstruct the data with 5mm slice thickness in a 512×512 image matrix and data was automatically transferred to MiStar for analysis. The CTA Circle of Willis – Carotids acquisition was obtained in the craniocaudal direction with 128×0.6mm collimation, 0.285 s rotation time, 1.2 Pitch, 120 kV tube voltage and 300mAs effective tube current time product. Contrast enhanced CTA images were acquired using 60 ml Omnipaque 300 (GE Healthcare, WI, USA), 30ml bolus sodium chloride and an injection rate of 5 ml/s. A soft spatial resolution kernel (i30f) was chosen to reconstruct the data with 0.6mm slice thickness in a 512×512 image matrix and data was automatically transferred to PACS. CTA scan range included superior to the anterior cerebral arteries and inferior to the left atrium. 284 patients at Christchurch underwent delayed cardiac CTA in addition to standard imaging due to a protocol change during the study period.

John Hunter: All patients were examined using a 384 (2 x 192) slice Dual Source CT scanner (SOMATOM Force, Siemens Healthcare, Forchheim, Germany or GE VCT Lightspeed, GE Healthcare, Waukesha, WI, USA). SOMATOM scan parameters for the CTP were using 192×0.6mm collimation, 0.25 s rotation time, 70 kV tube voltage and 450mAs effective tube current time product. Contrast enhanced CT perfusion scans were acquired using 50 ml Omnipaque 350 (GE Healthcare, WI, USA), 50ml bolus sodium chloride and an injection rate of 6 ml/s. A soft spatial resolution kernel (Hr36) was chosen to reconstruct the data with 5mm slice thickness in a 512×512 image matrix and data was automatically transferred to MiStar for analysis. The CTA Circle of Willis – Carotids acquisition was obtained in the caudocranial direction, with 128×0.6mm collimation, 0.25 s rotation time, 1.0 Pitch, 120 kV tube voltage and 350mAs effective tube current time product. Contrast enhanced CTA images were acquired using 50 ml Omnipaque 350 (GE Healthcare, WI, USA), 50ml bolus sodium chloride and an injection rate of 6 ml/s. A soft spatial resolution kernel (Bv36) was chosen to reconstruct the data with 1.0mm slice thickness in a 512×512 image matrix and data was automatically transferred to PACS and Syngo Via. CTA scan range included from aortic arch inferiorly to skull vertex superiorly. Additional scans through heart to assess LAA at 30 seconds and 2 minutes post injection were also performed from the carina to inferior to the left atrium.

**Supplementary Table 1:** 3-month mRS according to presence of left atrial appendage (LAA) thrombus.

| **Modified Rankin Scale (mRS)** | **Total (n=1435)** | **LAA present (n=58)** | **LAA not present (n=1377)** |
| --- | --- | --- | --- |
| 0 | 341 | 2 (3%) | 339 (25%) |
| 1 | 290 | 5 (9%) | 285 (21%) |
| 2 | 257 | 13 (22%) | 244 (18%) |
| 3 | 211 | 10 (17%) | 201 (15%) |
| 4 | 129 | 9 (16%) | 120 (9%) |
| 5 | 38 | 3 (5%) | 35 (3%) |
| 6 | 169 | 16 (28%) | 153 (11%) |

**Supplementary Table 2:** Subset of patients with atrial fibrillation (AF) taking anticoagulant prior to presentation and left atrial appendage (LAA) thrombus.

|  | Total with AF | AF with LAA thrombus present | AF with LAA thrombus absent |
| --- | --- | --- | --- |
| Anticoagulant at presentation | 134 | 11 | 123 |
| No anticoagulant at presentation | 305 | 35 | 270 |

**Supplementary Table 3:** Baseline demographic factors between cohorts.

| **Variable** | **Total (n=1435)** | **Christchurch Hospital** | **John Hunter Hospital** |
| --- | --- | --- | --- |
| Age median (IQR) | 74 (63-82) | 75 (66-83) | 73 (61-80) |
| Sex -female (%) | 589 (41%) | 346 (46%) | 243 (35%) |
| Hypertension | 825 (58%) | 444 (59%) | 381 (56%) |
| Diabetes | 301 (21%) | 158 (21%) | 143 (21%) |
| Dyslipidaemia | 405 (28%) | 215 (29%) | 190 (28%) |
| Atrial fibrillation | 439 (31%) | 261 (35%) | 178 (26%) |
| Ischaemic heart disease | 292 (20%) | 186 (25%) | 106 (15%) |
| Heart failure | 141 (10 %) | 81 (11%) | 60 (8%) |
| Peripheral vascular disease | 137 (10%) | 58 (9%) | 79 (11%) |
| Previous stroke | 252 (18%) | 160 (24%) | 92 (16%) |
| MLVO | 582 (41%) | 240 (32%) | 342 (50%) |
| LAA thrombus | 58 (4%) | 46 (6%) | 12 (1.7%) |
| Thrombolysis | 333 (23%) | 225 (30%) | 116 (17%) |
| MT | 333 (23%) | 165 (22%) | 168 (25%) |
| Any reperfusion treatment | 565 (40%) | 317 (42%) | 248 (37%) |
